# Phlebitis

**Published:** 1846-06

**Authors:** 


### PART VI.—SELECTIONS.

1. *Phlebitis.*—The internal surface of the whole vascular system, by which it comes in contact with the blood, is lined with a delicate serous membrane, which is the seat of several interesting diseases. That portion of it which lines the cavities of the heart, is liable to become inflamed from rheumatism and other causes, and thus is laid the foundation of most of the organic diseases of this organ. The lining membrane of the arteries is also the seat of inflammation in the disease called the dry gangrene, and in other cases, its inflammation causes the deposition of calcareous and cartilaginous matters which terminates in the production of aneurisms. The lining membrane of the veins is even more than the preceding liable to inflammation from mechanical injuries, surgical operations and other causes, and a train of morbid phenomena is thereby induced which has not been well understood until of late years.

The effects of Phlebitis are of entirely different character accordingly, as it terminates by adhesion, or passes on to suppuration.

### *Adhesive Phlebitis.*

It has been demonstrated by experiments on animals and by pathological observations, that whenever inflammation is excited in the lining membrane of any part of the vascular system, the blood has a tendency to become coagulated in the part which is the seat of the inflammation. In endocarditis, fibrinous concretions are very commonly found in the heart after death, and probably in many cases have a direct agency in causing death. In arteritis, the blood becomes coagulated in the veins, and in this way is produced the dry gangrene of the extremities in old people; and in other cases where the inflammation is acute and more extensive, a rapid gangrene of the whole limb occurs. Ollivier and others mention cases in which the pulmonary artery was inflamed, and death had been caused by the mechanical obstacle to the circulation opposed by the coagulum contained in the vessel. I shall presently allude to a case in which an inflammation of the venous sinus of the brain caused death by obstructing the circulation through this organ.

The first effect of Phlebitis, is then, to cause the formation of a coagulum which obstructs the circulation through the inflamed vein.

It is difficult to offer any explanation of this tendency of the blood to coagulate in an inflamed vessel. Indeed to do this, it would be necessary first to explain why the blood remains fluid while circulating in healthy vessels. This fluidity does not depend on its motion, nor on the exclusion of air, nor on any other mere mechanical or physical condition, for all these conditions may be maintained in blood which has been drawn from a living vessel, and yet it coagulates. The fluidity of the blood in the living body depends on some nervous influence exerted on it by the walls of the vessel, and accordingly it is found that where this nervous influence has been diminished, the tendency of the blood to coagulate, is increased. After blows on the head, the blood coagulates rapidly, as well as after hemorrhages; so that if in drawing blood from a patient, we receive it in separate cups, we find that the portion last drawn coagulates most rapidly on account of the tendency to faintness which the loss of blood has occasioned. Thus it is, that by an admirable provision of nature, the faintness, caused by the loss of blood, has a tendency to stop accidental hemorrhage, not only by diminishing the impulse of the heart, but also by the increased disposition of the blood to coagulate and thus plug up the bleeding vessel. Hence too, the utility of rapid abstraction of blood, so as to induce faintness, in cases of internal hemorrhage.

All that can be said in explanation of the coagulation of the blood in an inflamed vessel, is that it depends on a diminution or perversion of the nervous energy in the part.

I return from this digression, to trace the phenomena which follow this coagulation of the blood in an inflamed vein. Gradually the colouring matter is absorbed, so as to leave a fibrinous mass, which after a time becomes organized, and the vessel is thus converted into a solid cord. In other cases, when the inflammation is more limited, or less severe, the coagulum may be washed away by the current of blood, and the circulation through the vessel restored as before.

These are the changes which occur in what is called Adhesive Phlebitis. They are of little importance, and in general, give rise to no constitutional disturbance. However, besides these local changes, some other disturbances may occur which depend on the mechanical obstruction to the circulation caused by the obliteration of the veins. The extent of these secondary disturbances will depend on the size and situation of the vein which is the seat of the inflammation.

When the calibre of a small venus trunk is thus obliterated, the blood readily makes its way through collateral branches by reason of the free anastomoses existing in all parts of the venus system, and no notable derangement of the circulation ensues. But when large venus trunks are inflamed, and the inflammation extends to their ramifications, serious accidents may be produced by their obliteration. The impediment to the circulation causes a venus congestion in the part, and this is followed by tumefaction, serous effusion in the cellular tissue, and functional derangement of the organ effected. When an important organ is thus effected, death ensues.

One of the most frequent cases of Adhesive Phlebitis is seen in the disease called Phlegmasia Alba Dolens.

It has been remarked by Cruveilhier, that the uterus after labor is in the condition of a wounded organ, and inflammation of its veins invariably follows, and more particularly of those situated where the placenta was implanted. In the great majority of cases, this inflammation subsides after a few days, and gives rise to no functional disturbance, but in some cases it extends to the large veins of the pelvis, and thence to the crural veins and their ramifications of one side, and then we have the symptoms of Phlegmasia Alba Dolens.

*Suppurative Phlebitis.*

It is well known to surgeons, that certain conditions of the constitution, and certain external influences give to all wounds a strong tendency to pass to suppuration. Among these external influences are the effluvia of large cities, and still more of crowed hospitals, so that surgeons practising in such circumstances rarely see an amputation healing by adhesion. Now it is precisely in these conditions that Phlebitis has also a tendency to pass to suppuration, and then it gives rise to a train of results which I am now to describe.

When suppuration is about to occur in an inflamed vessel, it is preceded by the formation of a coagulum which obliterates its calibre. This coagulum is more dense at its surface where it is in contact with the vessel than in its central part, and hence, when suppuration commences in the walls of the vein, the pus passes by infiltration into the centre of the coagulum. If the vein is examined at this stage of the disease, there is found a purulent collection in the centre, surrounded by a fibrinous coagulum adhering to the walls of the vein, and forming, at either end, a dyke which prevents the mixture of the pus with the blood. As the collection of pus increases it may make its way through the vessel and point externally, and then we have a superficial abscess communicating with the cavity of a vein. Such abscesses, it would seem, are not very uncommon in surgical practice, though it is not always easy to determine their character. This disease is still local, and gives rise to no constitutional disturbance; it is not more serious than adhesive Phlebitis.

We now come to the case in which, the coagulum which plugged up the vessel, and separated the purulent deposite in its interior, from the blood, gives way and allows the mixture of pus with the blood. The occurrence of this accident is marked by the most serious symptoms, pointing to a termination almost invariably fatal. The patient, whose position up to this time had presented nothing alarming, is suddenly seized with severe chills, followed by typhoid prostration, and death occurs in from eight to fifteen days.

This is the *Purulent infection of the Blood.*

Examinations after death from this cause, show the inflamed veins filled with pus and sanguine concretions. This purulent matter can be traced along the veins towards the heart. Some have supposed that they had detected pus globules in the blood by a micoscropic examination, but there are some circumstances which render this proof of their presence in this fluid doubtful. M. Mandl has found in healthy blood, fibrinous concretions which might readily be confounded with pus globules. The most striking appearance in these cases is the great number of abscesses found in the lungs, liver, brain and muscles. Purulent effusions are also found in the joints. Dance has particularly described the appearance of these abscesses, and his description is important as indicating their mode of formation. He establishes three stages of their development. In the first stage, there is found a sort of ecchymosis or sanguineous infiltration, in the midst of, and around which, are found veins containing pus; the second stage consists of the formation of a nucleus which is hard and first of a blackish and afterwards of a whitish colour; finally, in the third stage, this nucleus softens and is converted into a purulent collection which at first occupies the centre, and then the whole of the engorged portion. These three stages are commonly seen in the same subject. Sometimes death takes place before any of these lesions have passed beyond the first stage. These abscesses have been called *Metastatic* from an erroneous view of their mode of formation.

The symptoms of the purulent infection of the blood have been very well detailed by Berard, as follows:

"It is announced by a violent *chill*, more or less prolonged. The feeling of cold is intense and the patients ask for more covering as in intermittent fever. This symptom had been noticed by good observers, who had recognized the danger of it before its true meaning had been discovered. Have you had chills? Such was the question which Dupuytren always addressed to those on whom he had operated. To this succeeds a marked heat and reaction, followed by a sweat which is viscous and not critical. The chills are renewed within twenty-four hours, and afterwards several succeed each other at intervals. It is only in the first days that the chills are intense; they are afterwards transient or even entirely wanting."

"The pulse is always very frequent, its other characters are variable; towards the end it becomes small and thready."

The patients are often delirious during the night. During the day, they are unconcious of the danger of their situation; they suffer little, and if questioned, answer that they feel well.

"At an advanced period of this disease, the skin takes a yellowish tinge as in jaundice, but the other signs of jaundice are wanting."

"The urine and other excretions are fetid; diarrhea is often present."

"Purulent collections sometimes form in the cellular tissue; at other times pus collects in some of the articulations."

"Sometimes there are symptoms and signs indicating the formation of abscesses in the viscera, but in general they are difficult of detection. The abscesses of the lungs are too small to give rise to any decided signs by auscultation, or to produce much flatness on percussion. They are accompanied by a slight cough and sometimes by rusty expectoration."

"When the metastatic abscesses have caused an inflammation of the serous membrane covering the organ in which they exist, it is then possible to detect the presence of this inflammation. Such cases are common."

"As to the progress of the disease it is generally rapid. Death occurs in eight, ten, or twelve days, but some resist the disease during several weeks. The rapidity of the accidents is in proportion to the quantity of pus which has passed into the veins. Sometimes at the end of twenty-four or thirty-six hours, there is an apparent improvement which is very apt to deceive."

It had been remarked by the older surgeons that after blows on the head the patient sometimes died after exhibiting the symptoms similar to those I have described, and that abscesses were found in the liver and other organs. They had also observed similar occurrences after surgical operations followed by suppuration. The numerous abscesses observed after death were ascribed to the absorption of the pus from the suppurating surface and its transfer and deposit in the different organs, and for this reason these were called metastatic abscesses. Other explanations more or less fanciful were offered, but I believe, that it is to Dance that the honor is due of having first pointed out the connection of these accidents with Phlebitis.

In large cities and still more crowded hospitals, it is very rare to find wounds uniting by the first intention; they almost invariably pass to suppuration. It is in such situations that it is so common to see patients die after amputations or even after much slighter operations, exhibiting the symptoms during life, and the lesions after death which I have described as belonging to the purulent infection of the blood. This train of accidents, leading almost always to a fatal termination, occurs with deplorable frequency in the hospitals of Paris after amputations, and though with less frequency, it happens in the practice of all surgeons.

"When," says Berard, "purulent infection succeeds to a surgical operation or accidental injury, the wound after being painful, suddenly changes its appearance; its edges fall in and the granulations on its surface become soft and flabby, and pale. The pus is serous and fetid; its quantity does not diminish sensibly during the two first days, but after this, it becomes less abundant. If a bone has been divided, its surface is dry and of a grayish colour."

In what manner does the blood become infected in these cases?

I have already said that the older surgeons accounted for the formation of the abscesses found after death, by supposing the absorption of pus from the wound, and its conveyance by the circulation to the different organs where it is deposited; so that, in this view, the pus found in the abscesses was produced by the wound. This opinion appeared to derive confirmation from the dryness of the wound in such cases. It must, however, be observed that this dryness does not occur until about two days after the first symptoms have manifested themselves, and hence it must be looked upon as a consequence and not as a cause of the disease. But there are other considerations which render such an explanation inadmissible.

When pus is examined under a microscope, it is found to consist of globules floating in a fatty albuminous fluid, or liquor puris. These globules are notably larger than the disks of the blood, and modern physiology has shown that there are no openings in the capillaries by which bodies of this size could be introduced. The absorption of pus unchanged is therefore impossible.

Indeed we do find that purulent effusions are sometimes removed by absorption, as in the case of some abscesses in the pleura. In such cases, the absorption gives rise to no constitutional disturbance, because the pus has not been absorbed until it has undergone a change, which has deprived it of its distinctive characters, and thus rendered its introduction into the blood harmless. First, only the fluid portion is absorbed, leaving the globules, which either remain as a solid mass, or are dissolved before they are absorbed.

The pus which infects the blood must then come from some other source than the suppurating surface. This source was first pointed out by Dance, and his views have been adop ed and carried out by Cruveilhier and other pathologists.

The veins around a suppurating surface are necessarily involved in the inflammation. In ordinary cases this inflammation is of the adhesive character, and hence no general disturbance is caused by it. But under miasmatic influences, and in certain states of the constitution, there is, as already stated, a tendency in all inflammations to run into suppuration. Hence, in these circumstances, the inflamed veins around the suppurating surface secrete pus, which passes into the mass of the blood, as in the cases of inflammations of the larger veins. There is therefore no essential difference between the mode of production of the purulent infection of the blood from a wound or suppurating surface, and that which follows the ligature or other injury of a large venous trunk. The difference consists in the mode in which the inflammation is caused; in the one case it is by direct violence, in the other case by contiguity to an inflamed part. Phlebitis necessarily exists around a suppurating surface, and accordingly as it is of the adhesive or suppurative character will it be harmless or fatal.

Where there are veins around a suppurating surface which from being situated in bony structure, or from their connexion with fasciae, remain patulus, this accident is most likely to occur; not because the pus is sucked in by the effect of atmospheric pressure, as has been said, but because the pus coming in contact with the lining membrane of their veins, excites inflammation in it. For this reason, surgeons who practise in hospitals are much more apprehensive of the results of operations in which bones are divided, as amputations, than of those which involve only the soft parts. This explains also the occurrence of this accident after blows on the head, which excite inflammation in the diploic veins of the cranium, and when this passes to suppuration, it leads to the purulent infection of the blood. Hence those abscesses of the liver which so much puzzled the older surgeons.

Surgeons practising in healthy localities have rarely occasion to witness this terrible disease, although no locality can procure an entire exemption from it, nor can any care always guard against it. It is the scourge of crowded hospitals, and causes a frightful mortality among the amputated and wounded.

There is yet another mode in which this accident may be produced. As has been before remarked, parturition is always followed by Phlebitis, which in ordinary cases, passes off without notice. In other cases, it extends to the veins of the pelvis and even of the extremities, and causes phlegmasia alba dolens. Again in other cases, in consequence of the bad air of hospitals, or other causes, this Phlebitis passes to suppuration, purulent infection of the blood follows, and then we have the most fatal form of puerperal fever.

Thus it is, that Phlebitis, whether it takes place in the larger veins from direct injury, or in smaller veins from their contiguity to an inflamed surface, or in the veins of the uterus after parturition, may terminate in suppuration, and produce the purulent infection of the blood. Surgeons had long been familiar with the fact, that death often occurred after amputations and similar injuries, and with the appearances presented by the organs after death, but their interpretation of the fact was defective. Modern pathology first traced these results to their origin in the inflammation of the veins.

One question still remains. Why does the passage of pus into the blood cause the symptoms and lesions I have described?

No satisfactory answer can be made to this question. Pus exercises a poisonous agency on the blood, but in what mode it operates, is uncertain. Some experiments of Cruveilhier made it probable that the numerous abscesses of the viscera may be excited by the stoppage of a globule of pus, in a capillary vessel, which produces an afflux of blood and congestion, which characterize the first stage of these abscesses. But death is not caused by these abscesses alone, for it sométimes occurs before they are formed, or at least while they are only in their forming stage.

Before concluding this subject, I would speak of the condition of the system which resembles the purulent infection of the blood, and which is often confounded with it, but which differs from it in its mode of production and in its result.

Every practitioner knows that an abscess may exist for months and give rise to no constitutional disturbance, so long as the access of air to its contents is prevented. This is the case, for example, with purulent collections depending on caries of the vertebræ, which descend and point in the pelvis or on the thigh. If such an abscess is opened, we find that after a few days the pus which issues from it becomes fetid, especially if it does escape freely, and there appears fever and other constitutional symptoms under which the patient sinks after a longer or shorter period. The condition has some resemblance to the purulent infection of the blood, but is essentially different from it. There is here no suppurative Phlebitis nor passage of pus into the blood, nor do we find after death the numerous abscesses which are characterestic of purulent infection.

The condition I am now describing is attributed by Berard to the absorption of putrid matters in contact with the suppurating surface. So long as this surface produces healthy pus, which readily passes off, no constitutional disturbance occurs; but when the pus remains in contact with the surface, until it becomes fetid, then the fever and other disturbances come on. Berard calls this the *putrid infection* of the blood.

It is often very important to distinguish between these two conditions, and in general the diagnosis is not difficult.

The fever, which is one of the most constant symptoms of putrid infection, is not accompanied by the violent chills which usher in and attend the purulent infection. When this fever is prolonged, it assumes the hectic type.

In putrid infection the digestive organs almost always are affected: the appetite is lost, the stools become loose, and ultimately a colliquative diarrhea sets in. The patients wear away, and become emaciated; the skin assumes a dingy appearance; they are irritable and sensitive; their countenance is expressive of suffering.

The two conditions differ also in their progress. Some persons have chronic suppuration with putrid absorption, and resist it during months and years. Purulent infection is always fatal within a few days: it never becomes chronic.

They differ besides in the result. Putrid infection can be cured, provided we remove the cause; that is, the presence of fetid pus in contact with the surface in suppuration, whether we do this by preventing the accumulation of the pus as its focus, or by removing the part diseased. Purulent infection is incurable, if we except one or two cases of reputed recoveries among the thousands which are fatal. There are no remedies against it, and where it has once occurred, it is of no importance what is done to the wound in which it had its origin.

No treatment is known for purulent infection of the blood. Something may be done in preventing the suppuration of the veins, but when the accident has happened, all our efforts to arrest a fatal termination will be fruitless. There are one or two cases in which recovery took place, but in these it did not seem to depend on the treatment pursued.—*Transactions of the Medical Society of the State of N. Y. Dr. Hun's Address.*

